# ‘We’re all in the same boat’: An Interpretative Phenomenological Analysis study of experiences of being an ‘expert’ during patient and public involvement within Child and Adolescent Mental Health Services (CAMHS)

**DOI:** 10.1111/hex.13183

**Published:** 2021-02-02

**Authors:** Samiran Idrees, Samantha Hartley, Jasmine Heath Hearn

**Affiliations:** ^1^ Manchester Metropolitan University Manchester UK; ^2^ Division of Psychology and Mental Health School of Health Sciences Faculty of Biology, Medicine and Health Manchester Academic Health Sciences Centre University of Manchester Manchester UK; ^3^ Pennine Care NHS Foundation Trust Ashton‐under‐Lyne UK

**Keywords:** CAMHS, change, experiences, policy, public and patient involvement

## Abstract

**Background:**

Patient and Public involvement (PPI) has rapidly evolved into a key component in shaping the delivery of health services. However, little is known about what it is like to participate in involvement procedures that include representatives of multiple groups and in the context of developing new interventions for Child and Adolescent Mental Health Services (CAMHS).

**Objective:**

This study explored participants’ experiences of PPI, following attending a ‘consensus conference’, during which their views were sought in relation to the development of a proposed staff‐based intervention and key questions about its design and implementation.

**Design:**

Qualitative, semi‐structured interview study.

**Setting and Participants:**

Six participants, including service users and various frontline clinical staff team members, who had experience of CAMHS were present at the consensus conference and then asked about their experiences of being involved via semi‐structured interviews. The data were analysed using Interpretative Phenomenological Analysis (IPA). Young people, carers and frontline staff have been involved in the design and implementation throughout the broader programme of work of which this study forms part, although these groups were not directly involved in the design and implementation of this paper.

**Results:**

Three key narratives were present: (a) Previous Experiences Driving Expectations, (b) ‘We are all in the same boat’ and (c) The Realization of Multiple Identities. The results suggest that PPI involvement is a complex process that may be driven by positive/negative expectations, but that individuals value learning about others and recognizing different perspectives while reaching shared goals in improving services.

**Discussion and Conclusion:**

This study demonstrates the complexity of experience that service users and clinical staff face when engaging in involvement activities in CAMHS. The findings demonstrate the value in engaging multiple stakeholder groups while also highlighting the importance of proper consideration of the procedures involved and facilitators of engagement.

## 1 INTRODUCTION

In the UK, patient and public involvement (PPI) has a vital role in shaping and improving the delivery of national health‐care services, with a commitment to empower both individuals and communities to play a greater role in bettering health‐care services.^1^, ^2^, ^3^, ^4^ Organizations such as INVOLVE advocate PPI as central to health‐care services research. It is argued that putting people at the heart of decision making and promoting continuous engagement between both the public and decision makers is crucial.^5^ This is supported by an ever‐growing commitment from funding bodies such as the National Institute for Health Research.^6^

Within research contexts, PPI has been defined as ‘research being carried out “with” or “by” members of the public rather than “to,” “about” or “for” them’.^7^ However, the scientific and policy literature regarding PPI and specifically who is involved is ambiguous. Both participation and the concept of the ‘expert patient’ overlap.^8^ By ‘public’, INVOLVE include potential patients, carers and people from organizations that represent people who use services. INVOLVE’s view is that members of the public with lived experience who have worked in health care and/or who gain research knowledge and expertise do not lose their lived experience; people can ‘wear multiple hats’. Particularly where they are identified as the ‘end users’ of an approach or intervention, it might be appropriate to involve frontline clinical professionals as ‘experts by experience’ in PPI. It is therefore important to engage a diversity of ‘experts by experience’ that might include frontline clinicians alongside those who use services, where this fits the research and development objectives.^9^ Although the term ‘PPI’ might not be the best fit in this context, focusing as it does on the patient and public, advice is that it is still applicable, until an alternative is developed.

PPI research within health services has an inclined focus on the adult population, especially around adult mental health.^10^, ^11^, ^12^, ^13^, ^14^, ^15^ It is likely that there are additional complexities when developing PPI in child and adolescent mental health services (CAMHS), due to the developmental, systemic, autonomy and power issues involved. Research has shown an unacceptable variation in the quality of children and young people's mental health provision across the United Kingdom, with many young people not knowing their rights and feeling like they do not have a genuine say in decisions made about their treatment.^16^ Given the lived expertise of those experiencing mental health problems have in handling their own lives,^17^ building an evidence base for innovative approaches in mental health services should be led as a merger of expertise between experts by experience and the expertise of the ‘professional’.^18^

Successful PPI engagement can often be impeded by differing ideas of the meaning of ‘involvement’ held by both professionals and service users.^19^ PPI within services tends to remain at the consultation level, with professionals’ decisions taking priority.^20^ Indeed, research has found that patients report feeling uncomfortable challenging health professionals’ opinions and practices,^21^, ^22^ with other barriers to shared decision making including lack of time, confidence or skills to fully involve patients as equal partners in care.^23^ This may be a particularly prominent barrier in child and adolescent mental health settings, as more often there is a parent/caregiver involved in their care. In addition, it is important to note that unlike adults, children often do not self‐refer to services, in turn creating a power imbalance between both adults and young people, highlighting the importance of examining experiences of PPI within different contexts, including factors that may hinder the development of care and participation.

Evidently, PPI is crucial for the outcome of health‐care services and there are gaps within the literature when exploring PPI in relation to CAMHS, where there might be additional considerations and complexities. Furthermore, attention has grown in the direction of understanding the key components of PPI and the impact this has on research. There is room to develop a wider understanding of PPI, particularly in CAMHS settings, considering the complexity of social processes in such services, and developing a richer understanding of the processes involved that could help facilitate successful PPI.^24^ This paper therefore investigated experts’ experiences of being involved in PPI within a CAMHS context, where the experts occupy different background groups; young people, carers and frontline clinicians.

### 1.1 Aims and research questions

This research aims to explore the experiences of those taking part in public and patient involvement within CAMHS. We aimed to explore:

- Individuals’ experiences of being involved in PPI within a CAMHS research and service development event, highlighting what might facilitate this to be effective in future studies.
- What it means to be an ‘expert’ within PPI for all parties involved, including young people, carers and frontline clinicians.

## 2 METHODS

### 2.1 Broader programme of work

The current study explored participants’ experience in the context of their involvement in the development of a new staff‐based intervention for CAMHS (https://osf.io/vr47z/). The intervention has been developed due to the paucity of evidence‐based forms of support in these area^25^ and therefore, the involvement of those with lived experience was crucial to deciding on key elements of the design and implementation. As the intervention is staff‐based, those defined as experts by experience included frontline clinicians, alongside young people and their family members. Representatives of these groups (12 in total) were invited to a ‘consensus conference’, during which their views were sought in relation to the proposed intervention. The consensus conference was held in person at a university location and was facilitated by the research programme lead (SH). This forum provided an opportunity to explore the experience of different groups of ‘experts’ participating in the same involvement process and thus provide insights into relevant processes and recommendations for similar future exercises.

### 2.2 Design

This study has implemented an exploratory, qualitative interview design, using IPA to analyse semi‐structured interview data gained through a small sample of individuals who are considered as an expert by experience within CAMHS services.

### 2.3 The selection of participants

IPA focuses on small, homogenous and purposive samples with direct experience of the phenomenon of study. The current study recruited participants who shared an experience of involvement in the same consensus conference, despite their differing backgrounds. Although there is no ‘right’ sample size,^26^ the typical sample sizes vary from one to fifteen^27^ and there are suggestions that research using IPA should wish for an expert sample that is small in numbers.^28^

Purposive sampling was used to select participants who were staff (nursing staff, doctors, psychologists), carers and recent service users of CAMHS. The main researcher of the current study (SI) was a bystander during the involvement process.

### 2.4 Data collection

Participants were interviewed over the 2 weeks following the consensus conference at locations convenient to the participants. Interviews were conducted mainly by SI (n = 4), and two were conducted by SH due to the availability of the participants. Only the interviewer and participant were present during each interview, and participants were aware of the interviewer's background and their relationship to the project. A semi‐structured interview schedule (see Table 1) was developed in consultation with the relevant population groups. Each interview was audio‐recorded using an encrypted dictaphone. Audio data were transcribed and anonymized.

**Table 1:** Interview schedule

Experiences before the PPI engagement event: What were your first impressions of being invited to attend the consensus conference? What did you think was being asked of you? What did you expect your role in the conference to be? What were your hopes for the conference? Did you have any worries about the conference? What were they? Who else did you think might be invited?
Experiences during the PPI engagement event: How did you find attending the conference? How would you describe your input during the discussions? How did you feel others’ responded? Do you feel your contributions made an impact? In what ways? Why? What were your thoughts and feelings during the conference discussions?
Experiences after the PPI engagement event: Can you describe your experience of being involved in the conference? What have you learnt about yourself during this conference? What have you learnt about being a part of a conference? How would you describe your interactions within the consensus conference? What, if anything, would you have changed about the experience if you could? Would you recommend any changes to future conferences?
Experiences of being considered an ‘expert’: Describe what being an expert means to you? Do you class yourself as an expert? If so why? If not, why not?

### 2.5 Analysis

Semi‐structured interviews offered an opportunity to acquire in‐depth first‐person accounts,^29^ facilitating the elicitation of stories, thoughts and feelings.^30^ Transcripts were analysed using Interpretative Phenomenological Analysis^l^ ^31^ (IPA), which was suited due to its ability to make sense of individual's experiences in specific contexts.

As IPA is an idiographic approach, it allows the researcher to explore the experiences of the participants, creating a rich and detailed understanding of the topic explored^32^ ^.^ IPA involves the researcher uncovering the participant's lifeworld through exploring their personal ‘lived experiences’.^30^, ^33^ IPA was selected as an appropriate approach in order to explore the ‘lived experiences’ of participants engaged in public and patient involvement based on their experiences of CAMHS services as opposed to simply an ‘experience’. An emphasis of experience is addressed by IPA in aiming to understand the context and meaningful world of individuals^34^ and the importance of the participants conveying how they are experts by experience.^30^

In observance with IPA’s idiographic obligations, each interview was analysed in‐depth on an individual basis.^30^ Each recording, listened back to at least twice, and the transcripts read several times. Initial annotations and phenomenological reduction were made, with exploratory comments describing initial thoughts about the content, language use and more conceptual comments in the wide right‐hand margin of the transcripts.^30^ Once each individual's experience had been analysed, commonalities and themes across participants were explored through cross‐case analysis.

SI led the initial analysis, with supervision from JH. JH, who was independent of the involvement process, helped validate interpretations and themes identified, offering a more ‘outsider’ perspective. The results were not specifically member‐checked with participants but were fed back to them and comments invited. A summary was also checked for accessibility with representatives of the same groups (clinicians, young people and carers).

## 3 RESULTS

Three themes central to participants’ experiences were developed. Participants’ descriptions acted as windows to their experiences, demonstrating the impact of prior expectations on their experiences, conveying a sense of connectedness with others involved in the PPI process, and the process of PPI engagement acting as a facilitator for the realization that each individual participant had multiple identities. The presence of themes within each participants’ account is highlighted in Table 2.

**Table 2:** Table of Themes

	Catherine (youth service user)	Ann (parent)	Sally (psychiatrist)	Daniel (support worker)	Rose (psychologist)	Farah (assistant psychologist)
Previous experience driving expectations	*	*		*	*	*
‘We are all in the same boat’	*		*	*	*	*
Realization of multiple identities	*	*	*	*	*	*

* Indicates presence of theme.

### 3.1 Theme one: Previous experience driving expectations

The first theme was expressed by several participants, highlighting their understandings of the potential of PPI to change health care based on their past experiences. Such experiences included when their provision of feedback was not acted upon within health‐care services previously, meaning that participants were unable to see whether and/or how their involvement had made any difference. Such past experiences consequently informed their attitudes and expectations towards PPI:

*I think it meant a lot, especially when you work in these settings, it [PPI] does get lost in like translation. You do feel like sometimes you are trying to express how you feel and stuff like that and sometimes it doesn’t get listened to, or you feel [said with emphasis] like it doesn’t due to all the different hierarchy levels and stuff, being passed down something like that*. (Daniel)

Daniel had been involved in PPI before and demonstrated feeling as though his previous experiences were inadequate, expressing a general concern of not feeling heard or whether his contributions would make a difference, with perceptions of being ‘overruled’ by services whose decisions are made by a hierarchy. Daniel and Rose found that they were pleasantly surprised and that their expectations were exceeded:

*I was a bit unsure prior to what my role would sort of be at first … I was pleasantly surprised, when it was more informal, just round the table like sharing and I thought it was really good*. (Daniel)

*I think initially I thought it would be more to facilitate a conversation and then it changed into something that I found helpful and useful… thinking about my own practice*. (Rose)

*I was like wait I need to think about this… this is important for me. I didn’t know it was but it is … and then when I was there I was really pleasantly surprised at just how useful it was to think about practice*. (Rose)

Both Daniel and Rose's expectations of PPI engagement were of it as a way of talking about a service generally, with little consideration of their role in PPI and how their personal practice might be influenced by their engagement. Rose described a realization mid‐way through, which led her to recognize the value of the event for her personal and professional practice and development. Both participants’ ‘pleasant surprise’ at ‘how useful’ the event was in for example stimulating thinking around professional practice also serves to demonstrate prior expectations of the event being something to contribute to but perhaps not necessarily learn from.

Prior expectations were also reflected by Catherine, who discussed concerns around not being involved in her own care:

*They should come speak with the young person and the family first before they make any difference. Because when I was [in the service] with like my care plan and stuff my mum wasn’t involved and it got done like half way through my admission, rather than at the start*. (Catherine)

*I thought it was quite good. Because I could get my point across so I wasn’t feeling left out. I knew I could input and made sure*. (Catherine)

With the overall health care she received being inconsistent, Catherine explains how involvement of both the service user and carer is vital, based on her experiences of being service user and at times feeling isolated from her own care. Catherine explains how her mother was not involved in her care until half way through the process, highlighting the inconsistency in her care. This subsequently left Catherine with concerns that her future contributions as a PPI expert may not be reflected in changes in health‐care systems. However, Catherine's actual experience reflected very differently to her expectations, and she expressed her enjoyment in giving voice to the voiceless, based on her experiences of being service user. Catherine felt as though the most important factor when making decisions about health care is involving the service user and giving them a voice and thus playing a bigger part in PPI.

It was clear across several participants the need to make a real influence and wider impact, recognizing patient and public involvement as a possibility to genuinely make a difference and influence decision‐making processes within CAMHS and therefore avoid being controlled by past organizational systems.

### 3.2 Theme two: ‘We are in the same boat’

The second theme concerned participants’ interpretations of PPI involvement and the people within it, which was often contradictory to their prior experiences and expectations. An essential feature of PPI was meeting other people in a similar situation, with comparable experiences and encounters:

*I kind of felt like well if they are in the same position as I am, then we are all in the same boat … I kind of felt equal, even though you’re like in a high position, like in a job. I kind of felt like we could all respect each other on like a level*. (Catherine)

Appreciation of being in similar situations and having a shared purpose enabled sharing experiences and provided opportunities for interpersonal learning and self‐reflection and modelling. It also facilitated a sense of community and group problem‐solving to reach shared goals that bolstered a shared, task‐motivated social identity which validated Catherine's experience.

Indeed, this was also reflected in the narratives of the health professionals involved in the PPI consultation:

*I didn’t feel like it was me vs them. The research was something I was interested in and it didn’t seem like separated or segregated, it felt very inclusive. Like I felt like we were all learning and sharing*. (Daniel)

*I’ve been to like a few before, that was… This was probably the one I’ve enjoyed the most … I don’t know why it was, maybe it’s because it’s something I’m passionate about. Just, I think it’s how open and welcome everyone was, I didn’t feel like there was a hierarchy, everyone was just speaking and sharing and helping each other sort of thing. I knew a lot of people were like asking about the pace training and stuff life that… everyone wanted to, like, better each other. It was really nice and refreshing*. (Daniel)

Developing supportive relationships within the consensus conference context was a vital element of participants’ experiences and supported the shared decision making that participants were involved in. Participants discussed their appreciation of the value of the varied perspectives alongside recognizing that they were all working towards the same goal:

*I have been a part of conferences before, I suppose it just sort of reaffirmed that as a doctor you might have a different perspective on things than other people in the multidisciplinary team. But, a sort of complimentary perspective I think … We all might have been looking at things in a different way but I think we all had the same aims and objectives*. (Sally)

Sally's discussion highlighted her prior knowledge of having varied perspectives, but also that her participation in the consensus conference ‘reaffirmed’ her appreciation of this. The group‐based involvement in the decision‐making process emphasized to Sally the value in such processes to provide a road map for improvements in services from a holistic perspective.

This is also evident with several participants, with the emphasis of belonging and being a part of something bigger, motivating continued involvement and participation:

*I guess that on most wards they have that something that they’ve thought about and wondered how they manage it. So I think it was quite nice, to see that it’s not just us that feel that way … I think it makes you an expert from your perspective, so you’re not an expert of the system as a whole but you are an expert of what you see, feel and witness on a day to day basis. Because I guess the nurse is going to have a different expertise around that from me, but we all have our own way, and all of that is one*. (Farah)

*I got a lot out of it in terms of seeing that everyone was pretty much in the same boat and I think that’s nice to be a part of. And it makes you stay motivated that you want to stay involved in that little group and keep going and come to the next one and stuff to see how things have evolved and adapted*. (Farah)

Farah's quotes demonstrate her perception of herself as an expert in her domain, and her acknowledgement and appreciation of the diversity of perspectives across an entire system. She echoed the perceived value in sharing insights across different stakeholder groups, such as nurses. Farah surmises that each single perspective, while holding its own unique value, feeds into a whole system, underscoring the need for consideration of all perspectives to develop optimal understanding and care delivery. Like Catherine, Farah uses the phrase ‘in the same boat’ and echoes her appreciation of learning that her experiences and goals are shared with others. This feeds into the development of a sense of community with the group, serving to motivate continued engagement in the process and to learn about how systems and processes have been adapted to meet their shared goals.

Ann discussed numerous roles from being a parent, to being *human* and what it may be like to be a caregiver and how important it is for these roles to be recognized during the PPI process:

*Well at the end of the day I’m a human being, I’m not going to like them, but they are health professionals, I’m a parent. It’s good for them to hear our side of what’s going as an inpatient as to what they are dealing with on a day to day basis*. (Ann)

Ann's quote reflects that once a part of PPI, there can be an acknowledgement that everyone's contributions are of value. Putting aside any judgements and perceptions of others, and recognizing that everyone is working towards the same end goal was key to feeding into the improvement of services; everyone was in the same boat.

### 3.3 Theme three: Realization of multiple identities

The third theme reflected the impact that engaging in PPI engagement had on the development and realization that participants had multiple identities above and beyond that of the ‘expert by experience’. This was a process echoed by both professionals and service users. Rose began her interview discussing her professional role and her initial view of others in the PPI process as patients, with her initial perception of her role as a facilitator of the discussion:

*I think in the beginning I suppose I was thinking about offering more of a psychological perspective than maybe other people that were there and I think it kind of shifted. I remember the shift, because I remember starting to think about 5 minutes in even … starting to think actually do I do this? And started to question myself and then I transitioned into more of like one of the people that felt like they were doing the reflecting and learning. Then I found it that I was more of an active participant*. (Rose)

Rose described quickly finding that her expectations of the PPI discussion were met with a contrasting reality, with a cognitive shift from a perception that perhaps it may not have been a helpful process for her personally to realizing that she could also learn from this process and contribute to the development of services. This demonstrates her ‘transition’ from perception of herself as an expert, interchangeably becoming an active learner within the process; PPI facilitating a flexibility in roles outside of the usual structures.

Farah also acknowledged her professional role within CAMHS as an assistant psychologist:

*I think before I got there, I was thinking I was a bit reluctant because you’re an assistant psychologist and you’re not qualified, so you do worry about what other people would be thinking and I guess that was my initial concern. And when I got there and saw it wasn’t just a bunch of qualified professionals and a mix of different people with first‐hand experience, I think that put me at ease, rather than it be like these are all professionals and there’s me… it wasn’t that dynamic*. (Farah)

Farah's experience was of hesitancy around the extent of her professional role and feeling ‘less qualified’ than other members of staff, which made the PPI experience somewhat daunting for her. Her realization that she was qualified in an additional, different way (in her first‐hand experience of the CAMHS service) was beneficial in normalizing the PPI process, addressing barriers in perceptions of expertise and hierarchy, and facilitating the sense of ‘ease’ and collaborative nature of the encounter.

As a service user, Catherine described her struggles with seeing past the identity of the health professionals involved in the PPI process.

*It’s weird, like it… I know you’re [the health professionals] a normal person and that but it’s just dead weird… It just is, like I know you’re a normal person and that but it’s just because of the job you do, it’s just weird*. (Catherine)

*I think they [the health professionals] would have felt the same as me, because I was the young person there and with a lot of professionals and other people you don’t know, [it’s] quite scary inputting, when they’re all professionals and there’s just you*. (Catherine)

Catherine's repetition of the PPI engagement with health professionals as ‘weird’ emphasizes her difficulty in seeing past the professional roles and relationships that she held with those professionals, which may have acted as a barrier to her disclosure of her experiences in PPI settings. Catherine also highlights the tension she experienced as a result of seeing herself as ‘just’ a service user, implying her concerns that she was alone in that experience and does not have the appropriate knowledge or experience to contribute. Additionally, the standard hierarchy that Catherine had experienced (as a service user working with health professionals) appeared daunting and difficult to overcome. She described the process of shifting from being a passive recipient of health care to an active expert by experience stance as ‘scary’, depicting the potentially challenging nature of PPI engagement. However, as Catherine settled into the PPI discussion, she found that this fear dissipated:

*It was quite good, because I could get my opinion out and be like, confident and be who I actually was, rather than just being quiet and sitting there*. (Catherine)

Catherine described feeling more settled in the PPI engagement after realizing that the process was collaborative and supportive in nature. As she was responded to helpfully and became more familiar with the process, the realization that she could be ‘herself’, and as such, owning her identity, as opposed to being ‘just’ the ‘service user’ helped to bolster her confidence in sharing her experiences and their impact on her wellbeing.

## 4 DISCUSSION

This study explored participants’ experiences of being involved as experts by experience in research and service development, from the perspectives of a variety of individuals within CAMHS. To our knowledge, this is the first study to explore the experiences of multiple types of experts by experience involved in the same involvement process and in a CAMHS context. The value and complexity of involving varied stakeholders is a key national priority^9^ and one that offers extensive opportunity for investigation, which the current study contributes to.

From the participants’ accounts, three key themes were discussed: (a) Previous experiences driving expectations, (b) ‘We are all in the same boat’ and (c) Realization of multiple identities. These findings demonstrate the extent of the impacts of being involved in the evaluation and development of services and research, reflecting implications that reached beyond service improvement and presenting multiple psychological complexities and benefits for the experts by experience who are involved in the process too. Such benefits included learning more about the process of PPI, having a supportive space to reflect upon and redefine previous expectations, and recognition that despite having different perspectives and roles all participants were working towards the same goals. Likewise, participants articulated a progressive realization that individuals can play multiple roles in PPI engagement by simultaneously facilitating the process, contributing their perspectives, learning about others’ experiences and reflecting on their own personal and professional practice. These findings demonstrate the value of involving experts by experience from different backgrounds/roles and that such involvement is a meaningful ingredient for successful PPI, as echoed in previous work.^35^

Participants often discussed their previous experiences of being involved in the evaluation and development of services, with their involvement in the present study offering them an opportunity to learn more about PPI and to reflect upon and redefine their expectations of such processes. This suggests a need to ensure that experts by experience are fully informed of what will be asked of their participation in order to manage expectations and ensure that participants feel able to contribute and are supported throughout the process. This also includes the integration of a feedback loop, whereby participants are able to see how their engagement feeds into service delivery, which can further bolster motivation to engage in PPI in future.^36^ Moreover, as previous experiences seem drive expectations, opportunities for individuals to explore their experiences and how these might be best supported should be offered outside of individual involvement activities in preparation; and services should consider how the quality of experiences in PPI and service delivery can either mutually support or degrade one another.

Participants discussed the value of meeting others with lived experience of services and echoed a sense of community with a shared purpose, even with multiple and varied perspectives. Participants’ accounts indicated that this sense of community bolstered participants in their task‐motivated social identity, which validated their experiences. This mutual support and sense of community is reflected in previous work examining the impact of PPI engagement on service users in (for example) lay researchers,^37^ and people with a diagnosis of schizophrenia,^38^ demonstrating a commonality across various health contexts. Such sense of support and community may also contribute towards the enhanced ability to problem‐solve in the management of health conditions.^39^ Likewise, literature suggests that shared decision making can improve knowledge, involvement, self‐confidence, self‐care and communication across families.^40^, ^41^ The present study supports this, with the addition of reflecting these benefits in different staff groups too. There might rightly be a reticence in involving multiple stakeholders in involvement exercises; a concern that PPI is best undertaken with specific groups separately. The current findings indicate that, where implemented effectively and with a sense of safety, bringing together experts by experience from different backgrounds might add value in terms of developing a sense of collaboration.

A novel finding in the current research is that of participants holding multiple identities, with the PPI process acting as a facilitator for this realization. Such co‐existing identities included facilitator, staff member, expert by experience, learner and human. Previous works suggests that some participants engaged in PPI may report unease at the changing roles between users and health professionals, for example shifting from a doctor–patient relationship to meeting as colleagues.^42^

Indeed, systematic review evidence suggests that challenges can occur when values and assumptions across PPI stakeholders do not mesh.^43^ However, this was not reflected in the present study, with participants articulating flexibility in adapting to their various identities in the consensus conference, and their appreciation for the views of others. This suggests that support and an open space to acknowledge and transition through various identities can provide value in the PPI process, which may reduce the risk of frustration and conflict between parties. Preparation could involve training all parties in PPI and allowing the time and space to develop trust and rapport between stakeholders, particularly within the CAMHS context, where there may be additional sensitivities to navigate in discussions. The consensus conference evaluated in the present study put an emphasis on all members being valued in their varied and multifaceted contributions and sought not to explicitly label these as coming from particular aspects of identity (all involved were invited to input to all aspects), which might have facilitated flexibility in role identity.

### 4.1 Strengths, limitations and Future Research

The current study is strengthened by its incorporation of multiple perspectives commenting on a shared process. The presence of the interviewer/s as an observer/facilitator at the consensus conference enabled consent processes to take place and familiarity to develop, which might have enhanced openness in interviews, although could have also encouraged demand characteristics. The interpretative and single‐interview nature of the present study means that the results reflect a single moment in time and do not reflect the developmental processes that might occur during a series of engagement events. Longitudinal interviewing by those independent of the involvement processes would aid the understanding of benefits and challenges experienced and the integration of participant feedback into service delivery. Further, the self‐selecting sample may mean that those with more positive experiences may have been more likely to take part in the study. The results, therefore, may not be reflective of those who experienced more challenges within the PPI process, and—importantly—those with expectations that led them to decline involvement. However, the purposive sampling strategy adopted in this study meant that commonalities within experiences were established and demonstrated the value in conducting PPI stakeholder engagement with a diverse group. Further work should explore how collaborative relationships across different stakeholder groups can be bolstered, to enhance participant confidence to continue to engage and to support the development and realization of the multiple potential roles that participants can play in PPI. Explicit evaluation of experience within different models of PPI would support clear recommendations regarding particular procedures that best facilitate the positive impact of involvement for researchers, clinicians and participants. It will be important for any future research to consider how to involve experts by experience not only as participants but as funded project leads and co‐investigators; the current study would no doubt have been improved by this.

## 5 CONCLUSIONS

This study demonstrates the various motivations, challenges and benefits experienced across service users, parents and staff members engaged in a CAMHS PPI exercise to contribute to clinical research and enhance service delivery. The findings demonstrate the value to all participants involved in such processes, and to the service concerned, in conducting such stakeholder engagement with a diverse group of perspectives.

## CONFLICTS OF INTEREST

Dr Samantha Hartley is funded by a National Institute for Health Research (NIHR) Integrated Clinical Academic Clinical Lectureship for this research project. This paper presents independent research funded by the National Institute for Health Research (NIHR). The views expressed are those of the author(s) and not necessarily those of the NHS, the NIHR or the Department of Health and Social Care. Dr Jasmine Hearn and Samiran Idrees declare no conflicts of interest.

## Data Availability

Shared data are not available for this study.

## References

[1] Department of Health . Healthy lives, healthy people: Our strategy for Public Health in England, vol. 7985. London: The Stationery Office; 2010.

[2] WHO . Primary health Care – Now more than ever. https://www.who.int/whr/2008/08_contents_en.pdf?ua=1. 2008 Accessed January 7, 2019

[3] KINGS FUND . Changing Relationships – findings of the patient involvement project. https://www.kingsfund.org.uk/sites/default/files/field/field_publication_file/changing‐relationships‐findings‐patient‐involvement‐project‐rosemary‐gillespie‐dominique‐florin‐steve‐gillam‐kings‐fund‐1‐october‐2002.pdf. Published October 1, 2002. Accessed January 2, 2019

[4] Tritter JQ , Lutfey K . Bridging divides: patient and public involvement on both sides of the Atlantic. Health Expect. 2009;12(3):221. 19754686 10.1111/j.1369-7625.2009.00566.x PMC5060499

[5] INVOLVE . Our Vison. https://www.involve.org.uk/?gclid=EAIaIQobChMIxeyolbG34AIVWYjVCh2Pswy0EAAYASAAEgIAJPD_BwE. Publication date unavailable. Accessed February 4, 2019

[6] National Institute for Health and Care Excellence . Patient and public involvement policy 2013. https://www.nice.org.uk/about/nice‐communities/nice‐and‐the‐public/public‐involvement/public‐involvement‐programme/patient‐public‐involvement‐policy. Publication date unavailable. Accessed January 18, 2019.

[7] INVOLVE .Frequently asked questions http://www.invo.org.uk/frequently‐asked‐questions/. 2018. Accessed October 3, 2019.

[8] Department of Health . Patient and Public Involvement in Health: The Evidence For Policy Implementation. London: Department of Health; 2004.

[9] INVOLVE . Different experiences. https://www.invo.org.uk/wp‐content/uploads/2020/03/Different_experiences_FINAL_edit.pdf. Publication date unavailable. Accessed May 25, 2020

[10] Cooper K , Gillmore C , Hogg L . Experience‐based co‐design in an adult psychological therapies service. J Mental Health. 2016;25(1):36‐40. 10.3109/09638237.2015.1101423 26670265

[11] Ekdahl AW , Andersson L , Wiréhn AB , Friedrichsen M . Are elderly people with co‐morbidities involved adequately in medical decision making when hospitalised? A cross‐sectional survey. BMC Geriatr. 2011;11(1):46. 21851611 10.1186/1471-2318-11-46 PMC3170190

[12] Pardon K , Deschepper R , Vander Stichele R , et al. Are patients’ preferences for information and participation in medical decision‐making being met? Interview study with lung cancer patients. Palliat Med. 2011;25(1):62‐70. 20621948 10.1177/0269216310373169

[13] Ocloo J , Matthew R . From tokenism to empowerment: progressing patient and public involvement in healthcare improvement. BMJ Qual Saf. 2016;25(8):626‐632. 10.1136/bmjqs-2015-004839 PMC4975844 26993640

[14] Rutter D , Manley C , Weaver T , Crawford MJ , Fulop N . Patients or partners? Case studies of user involvement in the planning and delivery of adult mental health services in London. Soc Sci Med. 2004;58(10):1973‐1984. 15020013 10.1016/S0277-9536(03)00401-5

[15] The Health Foundation . Helping people share decision making. https://www.health.org.uk/sites/default/files/HelpingPeopleShareDecisionMaking.pdf.2012. Accessed October 3, 2019

[16] Young Minds . About amplified. https://youngminds.org.uk/youngminds‐professionals/our‐projects/amplified/#about‐amplified. Publication date unavailable. Accessed January 9, 2019

[17] Faulkner A , Layzell S . Strategies for Living: A Report of User‐Led Research into People’s Strategies for Living with Mental Distress. London: The Mental Health Foundation; 2002.

[18] Faulkner A , Thomas P . User‐led research and evidence‐based medicine. Br J Psychiatry. 2002;180(1):1‐3. 11772841 10.1192/bjp.180.1.1

[19] Daykin N , Evans D , Petsoulas C , Sayers A . Evaluating the impact of patient and public involvement initiatives on UK health services: a systematic review. Evidence Policy J Res, Debate and Pract. 2007;3(1):47‐65.

[20] Barnes M , Coelho VS . Social participation in health in Brazil and England: inclusion, representation and authority. Health Expect. 2009;12(3):226‐236. 19754687 10.1111/j.1369-7625.2009.00563.x PMC5060493

[21] Aasen EM , Kvangarsnes M , Heggen K . Perceptions of patient participation amongst elderly patients with end‐stage renal disease in a dialysis unit. Scand J Caring Sci. 2012;26(1):61‐69. 21718340 10.1111/j.1471-6712.2011.00904.x

[22] Peat M , Entwistle V , Hall J , Birks Y , Golder S . Scoping review and approach to appraisal of interventions intended to involve patients in patient safety. J Health Services Res Policy. 2010;15(1_suppl):17‐25. 10.1258/jhsrp.2009.009040 20075123

[23] Edwards M , Davies M , Edwards A . What are the external influences on information exchange and shared decision‐making in healthcare consultations: a meta‐synthesis of the literature. Patient Educ Couns. 2009;75(1):37‐52. 19036550 10.1016/j.pec.2008.09.025

[24] Brett J , Staniszewska S , Mockford C , et al. Mapping the impact of patient and public involvement on health and social care research: a systematic review. Health Expect. 2014;17(5):637‐650. 22809132 10.1111/j.1369-7625.2012.00795.x PMC5060910

[25] Hartley S , Raphael J , Lovell K , Berry K . Effective nurse–patient relationships in mental health care: a systematic review of interventions to improve the therapeutic alliance. Int J Nurs Stud. 2020;102:102‐103. 10.1016/j.ijnurstu.2019.103490 PMC7026691 31862531

[26] Eatough V , Smith J . I was like a wild wild person: Understanding feelings of anger using interpretative phenomenological analysis. Br J Psychol. 2006;97(4):483‐498. 17018185 10.1348/000712606X97831

[27] Bramley N , Eatough V . The experience of living with Parkinson's disease: an interpretative phenomenological analysis case study. Psychol Health. 2005;20(2):223‐235.

[28] Hefferon K , Gil‐Rodriguez E . Interpretative phenomenological analysis. The Psychologist. 2011.

[29] Kvale S , Brinkmann S . Introduction to interview research. Doing interviews. 2007:2‐11.

[30] Smith J , Flowers P , Larkin M . Interpretative Pheneomological Analysis: Theory, Method and Research. London: Sage; 2009.

[31] Eatough V , Smith JA . Interpretative phenomenological analysis. Sage Handbook Qual Res Psychol. 2008;179:194.

[32] Smith JA , Osborn M . Interpretative phenomenological analysis as a useful methodology for research on the lived experience of pain. Br J Pain. 2015;9(1):41‐42. 26516556 10.1177/2049463714541642 PMC4616994

[33] Smith JA , Osborn M . Pain as an assault on the self: an interpretative phenomenological analysis of the psychological impact of chronic benign low back pain. Psychol Health. 2007;22(5):517‐534.

[34] Eatough V , Smith JA . ‘Interpretative Phenomenological Analysis’. The SAGE Handbook of Qualitative Research in Psychology. United States: SAGE; 2017: 193.

[35] Davis RE , Jacklin R , Sevdalis N , Vincent CA . Patient involvement in patient safety: what factors influence patient participation and engagement? Health Expect. 2007;10(3):259‐267. 17678514 10.1111/j.1369-7625.2007.00450.x PMC5060404

[36] Howe A , MacDonald H , Barrett B , Little B . Ensuring public and patient participation in research: a case study in infrastructure development in one UK Research and Development consortium. Prim Health Care Res Dev. 2006;7(1):60‐67.

[37] Newell C , South J . Participating in community research: exploring the experiences of lay researchers in Bradford. Community Work Fam. 2009;12(1):75‐89.

[38] Schneider B , Scissons H , Arney L , et al. Communication between people with schizophrenia and their medical professionals: a participatory research project. Qual Health Res. 2004;1494:562‐577. 10.1177/1049732303262423 15068580

[39] Oliver S , Milne R , Bradburn J , et al. Involving consumers in a needs‐led research programme: a pilot project. Health Expect. 2001;4(1):18. 11286596 10.1046/j.1369-6513.2001.00113.x PMC5060045

[40] Edwards A , Elwyn G . Inside the black box of shared decision making: distinguishing between the process of involvement and who makes the decision. Health Expect. 2006;9(4):307‐320. 17083558 10.1111/j.1369-7625.2006.00401.x PMC5060371

[41] Whelan T , Levine M , Willan A , et al. Effect of a decision aid on knowledge and treatment decision making for breast cancer surgery: a randomized trial. JAMA. 2004;292(4):435‐441. 15280341 10.1001/jama.292.4.435

[42] Hassey Dow K , Ferrell BR , Leigh S , Melancon CH . The cancer survivor as co‐investigator. The benefits of collaborative research with advocacy groups. Cancer Pract. 1997;5:255‐257. 9250084

[43] Brett JO , Staniszewska S , Mockford C , et al. A systematic review of the impact of patient and public involvement on service users, researchers and communities. Patient Patient Centered Outcomes Res. 2014;7(4):387‐395. 10.1007/s40271-014-0065-0 25034612

